# Two-component signal transduction systems in oral bacteria

**DOI:** 10.1080/20002297.2017.1400858

**Published:** 2017-11-27

**Authors:** Renata O. Mattos-Graner, Margaret J. Duncan

**Affiliations:** ^a^ Department of Oral Diagnosis, Piracicaba Dental School, State University of Campinas – UNICAMP, São Paulo, Brazil; ^b^ Department of Oral Medicine, Infection and Immunity, The Forsyth Institute, Cambridge, MA, USA

**Keywords:** Oral bacteria, two-component transduction systems, gene regulation, virulence genes, biofilm formation, *Porphyromonas gingivalis*, streptococci

## Abstract

We present an overview of how members of the oral microbiota respond to their environment by regulating gene expression through two-component signal transduction systems (TCSs) to support conditions compatible with homeostasis in oral biofilms or drive the equilibrium toward dysbiosis in response to environmental changes. Using studies on the sub-gingival Gram-negative anaerobe *Porphyromonas gingivalis* and Gram-positive streptococci as examples, we focus on the molecular mechanisms involved in activation of TCS and species specificities of TCS regulons.

## Introduction

How bacteria respond to environmental changes is regulated, in part, by two-component signal transduction systems (TCSs). These systems have been studied extensively in pathogens and environmental organisms with the goal of deciphering the activating signals and the genes induced in the response, i.e. the regulon of each system. Such studies include both classical and high-throughput technologies ranging from bacterial genetics to next-generation sequencing. In this review, we use studies on two well-known oral bacteria, *Porphyromonas gingivalis* and *Streptococcus mutans*, to illustrate the approaches used to understand how their TCSs work and the genes they regulate. Furthermore, strain- and species-specific variations have been discovered as well as alternate strategies that have evolved to compensate for gene loss.

## Oral microbiota

The generic term ‘oral microbiota’ refers to a large collection of different microbial communities whose development, maturation, and control are modulated by a wide range of environmental conditions. Shedding oral mucosa and hard tooth surfaces formed by enamel and/or dentin present different receptors for microbial adhesion, particular biophysical conditions (e.g. pH, temperature, moist, oxygen tension), as well as exposure to diverse host defence factors [1,2]. Dental biofilms contribute to robust microbial communities organized in a complex extracellular matrix that not only provides a structural scaffold but also protects the biofilm organisms from host and exogenous antimicrobial functions [3,4]. As in most biofilms [5], the composition and structure of the extracellular matrix of dental biofilms are dynamically modulated by the colonizing bacteria in response to local biophysical conditions and the availability of substrates that support its synthesis and other metabolic functions [6]. The biophysical and immunological challenges that affect biofilm formation on oral surfaces are site-specific. Micro-organisms in both supra- and sub-gingival plaque are exposed to differing temperatures, pH, redox potential, and availability of nutrients from diet, blood, or tissue fluids, as well as host cells and molecules present in saliva and serum [2,3]. Supra-gingival biofilms must adapt to fluctuations in nutrient availability in saliva, higher redox potentials, and oxidative stress, as well as host defence functions present in whole saliva and, to a lesser extent, in the serum-like gingival exudate (gingival crevicular fluid: GCF). On the other hand, sub-gingival biofilms depend less on saliva for nutrients than on the flow and composition of the GCF, and are adapted to low redox potentials and anaerobiosis [2,3]. Both supra- and sub-gingival biofilms may become dysbiotic under certain environmental and host conditions, which can promote dental caries or periodontal disease, respectively. The shift to a dysbiotic community involves changes in physiologic functions of the community members in response to the environmental conditions. To survive in such continuously changing oral environments, micro-organisms use regulatory systems to sense and rapidly respond to stimuli derived from other members of the microbial community, host functions, and exogenous factors, as addressed in the sections below. A major challenge is to identify the signals and functions that account for shifts in microbial communities from homeostasis to dysbiosis.

This review focuses on two members of the dental biofilms, *S. mutans* and *P. gingivalis*, because these species are consistently involved in dysbiotic processes associated with dental caries and periodontal diseases [7–9]. In part, because these species are relatively easy to grow and manipulate genetically, studies on these two oral pathogens led to the discovery of sophisticated mechanisms of virulence, several of which seem to be expressed by a limited group of species. Identification of these virulence functions is compatible with the notion that pathogens can disturb microbial-host homeostasis and cause infections depending on the host and environmental conditions [10–12]. For example, *P. gingivalis* expresses gingipain cysteine proteinases that subvert host immune functions and promote dysbiosis, giving rise to claims that it is a ‘keystone’ pathogen [13]. On the other hand, *S. mutans* expresses specific types of glucosyltransferases for the production of a stable glucan biofilm scaffold from sucrose, which favours the assembly of three-dimensional acidic microenvironments within the biofilm matrix, and the emergence of aciduric and acidogenic species, which characterizes cariogenic biofilms [14]. However, the expression of genes for virulence and metabolic adaptation to stresses promoted by dysbiotic processes are not constitutive, but modulated by the environmental stimuli. As we will address through this review, most of the genes involved in virulence of *S. mutans* and *P. gingivalis* are controlled through regulatory signal transduction systems, which allow microbial cells to sense local conditions and to respond readily to specific signals by altering their gene expression profile. Some of these responses may promote dysbiosis and increase the pathogenic potential of the microbial community. Therefore, defining the environmental signals that activate bacterial TCSs is an important step in order to understand the molecular mechanisms involved in the pathogenesis of biofilm-associated oral diseases.

## Bacterial signal transduction systems

In nature, bacteria are subjected to changes in local pH, osmotic pressure, temperature, redox potential, nutrient availability, and exposure to toxic chemicals. In order to survive these challenges, bacteria must be able not only to communicate with each other but also to perceive and respond to environmental signals. To cope successfully with all these selective pressures, bacteria have evolved simple but highly efficient signal transduction systems to regulate gene expression to match the specific challenge. Bacteria utilize three major classes of signal transduction systems: (1) one-component systems; (2) TCSs; and (3) phosphorelay systems [15]. One-component systems respond to intracellular signals whereby a cytosolic protein binds to a cytosolic ligand and undergoes a conformational change that allows binding to the promoter region of a target gene; such binding results in either activation or repression of gene expression. TCSs are primarily (but not exclusively) involved in adaptation to external stimuli. Each system is formed by a pair of proteins, a transmembrane sensor protein, and a cytoplasmic response regulator (RR). The sensor protein is phospho-activated in response to a specific environmental trigger, and then in a subsequent phosphorelay the activation signal is transferred to a cognate intracellular RR. The regulons of each TCS are variable and may include one operon to hundreds of genes [16]. The third signal transduction system could be defined as a variant of the TCS in the sense that the activation of the RR by the sensor protein involves multiple steps of transference of the phosphate group to intermediary proteins [17]. Additional phosphate signalling activities with similarities to eukaryotic pathways have been detected in prokaryotes including *P. gingivalis* [18,19].

TCS and phosphorelay systems are mostly abundant in prokaryotic organisms, but are also present in Archaea, and in some eukaryotic organisms [20]. However, the TCSs of eukaryotes are involved in more complex signalling cascades, likely evolved in function of the large size and complex intracellular structure [15,17]. Bacterial species can express several different types of TCS, which differ in structure depending on the types of signals sensed (normally chemical ligands) and in the set of target genes affected. The numbers of TCSs expressed by a species appears to correlate with the size of its genomes and more interestingly with the extent of diversity of the natural niches [21,22]. Table 1 shows the numbers of TCSs expressed for some to the most common bacterial species present in supra-gingival and sub-gingival biofilms. These TCSs could be predicted by sequence homology based on their domain architecture and on its genetic organization. Most of the genes encoding a TCS sensor protein (a histidine kinase, HK) form an operon with genes encoding its cognate RR and sometimes other accessory component. There are, though, exceptions in that an HK-encoding gene is not associated with a corresponding RR gene, or vice versa. These solitary HKs or RRs are also known as ‘orphan’ components. As observed with obligate intracellular species, e.g. *Mycoplasma* spp. [22], oral species that are mostly restricted to intracellular compartments of the host and/or to anoxic conditions, e.g. *Actinobacillus actinomycetencomitans* and *P. gingivalis*, have a limited number (two to seven) of complete TCSs (Table 1). On the other hand, bifidobacteria and streptococci, which are adapted to diverse oral sites, including different mucosal sites and supra-gingival or sub-gingival biofilms, harbour 11–15 different and complete TCSs (Table 1). Interestingly, *Tannerella forsythia* and *Treponema denticola* harbour a higher number of orphan HKs and RR than complete TCS, and evolutionary analysis of these genes might help to explain their biological significance. Although Table 1 includes representative strains of selected species, the number of TCSs may vary between strains of a species. As for example, the total number of TCSs identified in the genomes of *S. mutans* strains varies from 12 to 14 [23], and certain TCSs are less prevalent, while others are highly conserved. For example, the TCS ScnKR, likely involved in the regulation of bacteriocin production, is absent is a sub-set of *S. mutans* strains [23]. Molecular studies of several of these TCSs in particular *S. mutans* strains were previously reviewed [24]. Evolutionary analyses of TCSs indicate that bacterial lineages might acquire novel TCSs by gene duplication and lateral gene transfer under specific selective pressures. These ‘novel’ TCSs evolve to assume specific functions, likely to avoid unsuitable cross-talk with other pathways [22].

**Table 1.** TCSs in the genomes of oral bacteria.

		No. of complete TCS	No. of orphan HK, HHK, RR, HRR
Species and strain	Genome size (bp)	p2cs^a^;MiST2^b^	p2cs; MiST2
*Aggregatibacter actinomycetemcomitans*, D7S-1	2,309,073	2;2	13 (8HK, 5RR);
			6 (2HK, 3,1HHK)
*Bifidobacterium longum* ATCC 15,697 (JCM 1222)	2,828,958	14;NA	16 (8HK, 8RR);
			NA
*Fusobacterium nucleatum* subsp. *Nucleatum* ATCC25586	2,268,272	6;6	3 (1 HK, 2 RR);
			4 (2HK, 2RR)
*Lactobacillus acidophilus* NCFM	1,993,560	8;6	0;
			2 (2RR)
*Porphyromonas gingivalis* W83	2,343,476	4;4	4 (2HK, 2RR);
			4 (1HK,2RR,1HHK)
*Porphyromonas gingivalis* ATCC 33277	2,354,886	3; 3	5 (1HHK, 1HK, 3RR);
			5 (1HK,1HHK, 3RR)
*Prevotella intermedia* 17	2,119,790	2;4	9 (5 HK, 4 RR); 7 (3HK, 2HHK, 2RR)
*Rothia dentocariosa*, ATCC 17931	2,506,025	6;8	6 (3 HK, 3 RR);
			3 (2HK, 1RR)
*Streptococcus gordonii* Challis	2,196,662	14;13	3 (1 HK, 2 RR);
			4 (1HK, 3RR)
*Streptococcus mitis* B6	2,146,611	15;13	2 (1 HK, 1 RR);
			4 (1HK, 3RR)
*Streptococcus mutans* UA159 (serotype *c*)	2,032,925	14;12	1 (1 RR);
			3 (1HK, 2RR)
*Streptococcus mutans* LJ23 (serotype *k*)	2,015,626	13;12	1 (1 RR);
			2 (2RR)
*Streptococcus oralis* Uo5	1,958,690	11;9	1 (1 RR);
			4 (1HK, 3RR)
*Streptococcus salivarius* CCHSS3	2,217,184	13;11	6 (2 HK, 4 RR);
			6 (6RR)
*Streptococcus sanguinis* SK36	2,388,435	14;13	1 (1 RR);
			2 (2RR)
*Tannerella forsythia* ATCC 43,037	3,405,521	7;8	13 (8 HK, 5 RR);
			8 (1HK, 5HHK, 2RR)
*Treponema denticola* ATCC 35,405	2,843,201	5;3	10 (6 HK, 4 RR);
			11 (4HK, 1HHK, 5RR, 1HRR)

Data obtained from ^a^p2cs database URL:http://www.p2cs.org/ (Barakat M, Ortet P, Whitworth DE 2011 P2CS: a database of prokaryotic two-component systems. Nucleic Acids Res.39 (Database issue):D771-6. doi: 10.1093/nar/gkq1023) and ^b^ Mist2 database (URL:http://mistdb.com) (Ulrich LE and Zhulin IB. The MiST2 database: a comprehensive genomics resource on microbial signal transduction. 2010 Nucleic Acids Res. 38 (Database issue):D401-7. doi: 10.1093/nar/gkp940.).

## How TCSs work

**Figure 1.**
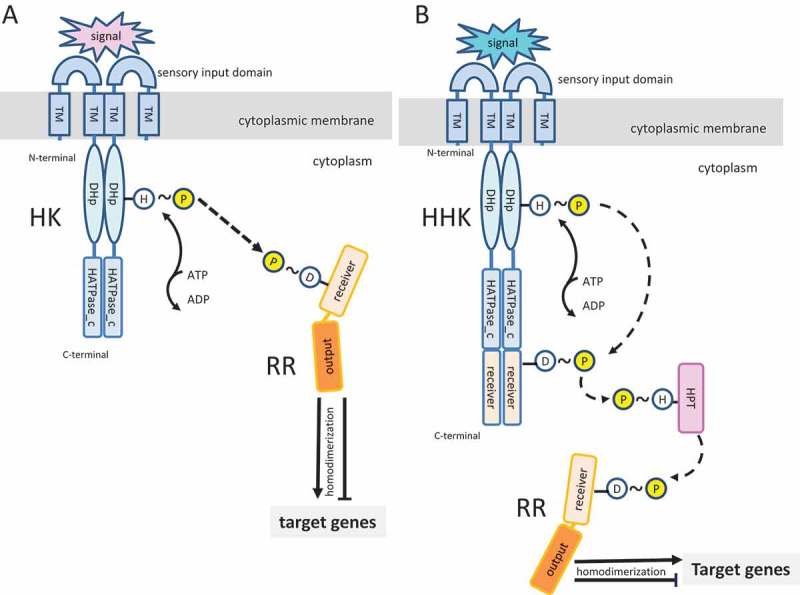
Schematic representation of histidine kinases (HK) and cognate response regulators (RR) of two-component systems (TCS). (A) Basic TCS consisting of a homodimeric HK with N-terminal extracytoplasmic sensory input and transmembrane (TM) domains. The cytoplasmic C-terminus contains the conserved DHp (dimerization and histidine phosphotransferase) and catalytic and ATP-binding (HATPase_c) domains. Although not shown in this simplified diagram, a variable number of cytoplasmic accessory domains of different classes may also be found in HK. Upon recognition of a specific environmental signal by the sensory domain, the HK undergoes autophosphorylation through HATPase_c-mediated transference of a phosphate group from ATP to the conserved histidine (H) in the DHp domain. This phosphate group is then transferred to a conserved amino acid (frequently an aspartate; D) in the receiver domain of the RR. This phosphorylation promotes conformational changes, frequently homodimerization, to permit interactions of the RR output domain with the regulatory regions of target genes. These interactions may activate or inhibit gene transcription. (B) Example of a hybrid HK (HHK), involved in more complex phosphorelay systems. In the schematic diagram, the HK contains a C-terminal receiver domain that receives the phosphate group and is involved in a phosphotransfer to the RR via a histidine phosphotransferase (HPT) intermediate.

The basic structure of a typical TCS consists of a transmembrane sensor protein, normally an HK with an extracellular (or membrane embedded) sensor domain and a conserved intracellular kinase core [16,17]. An external signal recognized by the sensor domain promotes ATP-dependent autophosphorylation at the histidine (H) residue of the kinase core. The phosphate group is then transferred to the cognate intracellular RR at its regulatory domain, normally to an aspartate residue (D), promoting conformational changes. This activated RR then binds to the regulatory regions upstream to the promoters of its target genes, inducing or repressing their transcription. HKs may also have phosphatase activities, to modulate the phosphorylation level through their cognate RR. Figure 1 illustrates two types of TCS, with basic architectural domains of the HKs and cognate RR. Most of the HKs are homodimeric proteins containing multiple functional domains linked by flexible hinges. The N-terminal sensor domain (input domain) is the most variable, since it defines the specificity for the environmental ligand and includes one to several transmembrane domains (TMs). The highly conserved dimerization and histidine phosphotransferase domain (DHp, also known as HisKA) is contained in the intracellular compartment and is responsible for autophosphorylation at the conserved His residue and for the phosphotransfer reactions (the transmitter domain). Next to the DHp is the conserved C-terminal catalytic and ATP-binding domain (HATPase-c; histidine kinase-like ATPase C-terminal domain), typical of several ATP-binding proteins. In addition to this basic domain architecture, HKs vary in the number and types of accessory domains (normally located between the input and the DHp domains), which assist in the transmission of the input signal and sometimes detect intracellular stimuli. Some of the common accessory domains are PAS (a domain common in Per-Arnt-Sim proteins), GAF (a domain common in cGMP-specific phosphodiesterases, adenyl cyclases and the bacterial transcriptional regulator FhlA), and HAMP (a domain common in HKs, adenyl cyclases, methyl-accepting proteins and phosphatases) [25]. The conserved domain architecture of RR includes the N-terminal receiver domain (REC) containing a conserved D residue that receives the phosphoryl group required for activation of the regulatory domain (output domain). In addition to HKs containing the sensor and transmitter domains (Figure 1(a)), there are hybrid HKs (HHKs). As exemplified in Figure 1(b), the HHK has a receiver domain for intermediary phosphotransfer (histidine phosphotranferase-mediated) before final relay to the receiver domain of the cognate RR. Some HHKs (called unorthodox HKs) also contain a histidine phosphotransferase domain (HPT). The P2CS database classified HKs into three major families, classical HKs, HHKs, and unorthodox HKs, as well as the CheA-like HKs [22,26]. There are also hybrid RRs (HRRs) containing HK domains [27,28]. There are 35 different families of RRs based on different types of output domains [22,26,29]. Approximately 50% of the activated RRs undergo homodimerization involving conserved residues at their receiver domains, and dimer formation is required for interaction with the regulatory regions of the target genes, which may contain tandem and inverted repeats [16,22].

Although sequence analyses of bacterial genomes reveals a diverse array of TCSs based on their conserved domain architecture, there is still limited information about their biological functions, and few studies have identified the stimuli responsible for HK activation [29]. Experimental studies and comparative genome analysis indicate that arrangement and topology of the N-terminal sensor and TM domains of HKs correlate with the type of stimuli sensed [30]. Based on the analyses of close to 4500 HKs, these sensor proteins were classified into three major groups. The first group includes HKs with an extracellular input domain, which is flanked by at least two TM domains. Basic HKs (Figure 1) fall into this large group and are involved in sensing extracellular (or periplasmic) stimuli, including nutrients and other soluble chemicals. The second group is characterized by N-terminal transmembrane regions formed by two to 20 membrane-spanning domains linked by short sequences, and thus with no clear extracellular sensor domain. This group seems to be involved in sensing stimuli directed to the membrane sites, e.g. membrane stresses, membrane transport and electrochemical gradients, or stimuli derived from other membrane components. HKs of TCSs involved in quorum-sensing in Gram-positive bacteria are within this second group. The third group of HKs is characterized by input domains at the cytoplasmic compartment. Examples of stimuli sensed by this group of HKs include cytoplasmic solutes or signalling proteins, stimuli transmitted through transmembrane proteins, or membrane-diffusible compounds [29]. Domain architectures of N-terminal and C-terminal regions of HKs of these three groups have been used to classify HKs of the three major groups into a relatively small number of sub-groups [29]. Few representatives of these sub-groups were functionally characterized, highlighting the need of functional studies on TCS. Some of these sub-groups analysed in oral bacteria are addressed in the next sections.

Because several bacterial species express a large number of different TCSs, it is important to understand how a particular RR is only activated by its cognate HK and no other HK. Several molecular and genetic studies have addressed this question [22,30,31]). There is evidence that each HK has a preferred affinity to its cognate RR, because the specificity of the HK–RR interaction involves the hypervariable regions flanking the conserved aspartate of the RRs. Mutations on these hypervariable regions are shown to affect the efficiency of interaction between a specific HK and the cognate RR [30,31]. A second factor affecting the specificity of the HK–RR interaction is the ratio of HK and RR production in the cell in response to a certain stimulus. Most of the operons encoding TCSs are self-regulated by the encoding TCS, implying that activation of an HK by its specific stimuli will also enhance the expression of this HK and RR [30]. In addition, because the genes encoding HK and RR of a specific TCS are expressed from the same operon, appropriate ratios of HK and RR production result in efficient interaction between these proteins. Specificities of HK localization at the cell membranes appear to avoid contact with HKs of other TCS. For example, some HKs are preferentially localized at cell poles, while others are restricted to other cell-surface regions, potentially avoiding physical interactions between non-cognate partners. Finally, because HKs commonly have phosphatase activity to dephosphorylate the cognate RR, they can control non-specific phosphorylation reactions by other TCSs or by low-molecular-weight cytoplasmic phosphoryl donors (e.g. acetyl phosphate; AcP) in the absence of the specific stimulus. Thus, there are several mechanisms by which cross-talk between different TCSs is avoided, ensuring that each TCS is activated only by its specific stimulus. On the other hand, because bacteria may need to respond to multiple signals simultaneously, the activities of multiple TCSs have to be integrated, and mechanisms of cross-talk between different TCSs and other transcriptional regulators are also required [30,31]. As addressed below, there is increasing evidence of TCS interactions with different classes of kinases and phosphatases, such as eukaryotic-like serine/threonine and low-molecular-weight kinases and phosphatases.

According to the predicted model of oral biofilm formation, streptococci are substrate-adhering pioneer bacteria, fusobacteria are bridging organisms, and *P. gingivalis* is a late colonizer that was shown by direct imaging to bind to both *Streptococcus* and *Fusobacterium* [32]. A series of studies examined proteomic and gene expression of *P. gingivalis*, *Streptococcus gordonii*, and *F. nucleatum* in a simulated biofilm in which resting cocultures were incubated for 18 h under anaerobic conditions [33–35]. While the proteome of *P. gingivalis* generally reflected its non-growing state, the expression of haemin uptake protein HmuR was upregulated during coculture, and an *hmuR* mutant showed decreased survival in the community [33]. Furthermore, *P. gingivalis* appeared to have a strong species-specific ‘dominant’ effect on the proteome of *S. gordonii*. It was suggested that the increased oxidative stress response of *S. gordonii* may protect *P. gingivalis* from secreted peroxide [34]. In addition, asaccharolytic *P. gingivalis* appeared to reduce glycolysis in *S. gordonii* and *F. nucleatum* [35] possibly to avoid production of acids that could inhibit *P. gingivalis* metabolism and or growth. A more recent RNA-seq analysis of *P. gingivalis–S. gordonii* coculture showed increased expression of genes in the *P. gingivalis* OxyR and RprY regulons, consistent with protection against peroxide produced by *S. gordonii* [36].

## *P. gingivalis* FimSR TCS

*P. gingivalis* fimbriae are associated with biofilm formation [37] and binding to other bacteria [38]. Production of fimbriae is regulated by the FimS-FimR TCS encoded by PGN_0904-PGN_0903 in strain ATCC 33277, and PG 1432-PG1431 in strain W83. In ATCC 33277, expression of *fimA*, encoding the fimbrilin protein subunit of fimbriae, is positively regulated by the FimR RR, i.e. it is an activator of *fimA* expression. It was demonstrated that FimR controlled the expression of several genes including five clustered around the *fimA* locus. Gene-expression analyses of mutant strains revealed a transcriptional cascade with FimR activating expression of the first gene of the cluster that encodes a key regulatory protein [39]. In addition, comparative analyses of fimbriate type strain ATCC 33277 and fimbria-deficient strain W83 revealed differences in their *fimS* loci encoding the FimS HK. FimS from W83 is non-functional because of a defective kinase domain that has a truncated conserved G3 box motif and so is unable to bind ATP for phosphorylation. Thus, in spite of possessing a *fimA* gene, strain W83 does not produce a *fimA* transcript or a FimA protein. Introduction of the functional *fimS* gene from 33,277 restored production, but not polymerization, of endogenous FimA subunits in W83 [40]. Recent work from Nishikawa focused on mutant analysis of the FimS periplasmic sensor region that contains eight tetratricopeptide (TPR) repeat regions. The TPR motif consists of up to 16 tandem-repeats of 34 amino acid consensus residues and mediates the assembly of multiprotein complexes and protein–protein interactions, implying that the environmental signal for the FimRS TCS might be a protein [41].

## RR RprY

In *P. gingivalis*, RprY (PGN_1186 in ATCC 33277, PG1089 in W83) is an ‘orphan’ RR because the usual close genetic linkage to a candidate cognate HK gene is absent. Using several experimental approaches (direct DNA-RprY binding screens, ChIP-on-Chip, and electromobility shift assays) it was established that the RprY regulon included genes associated with oxidative stress, iron transport, and sodium translocation [42]. RprY bound to the promoter of the first gene (*nqrA*) in an operon encoding Na**^+^**-translocating NADH: ubiquinone oxidoreductase, a sodium pump possibly involved in energy production by *P. gingivalis* from anaerobic respiration [43]. Indeed, RprY also bound to the *nqrA* promoters from *Bacteroides fragilis* and *Vibrio cholera* [42]. In the latter, NQR enhances cholera toxin production and is involved in generating intracellular superoxide [44,45].

The role of sodium (Na^+^) depletion as a potential environmental signal to activate RprY function was further investigated [46]. In *Escherichia coli*, an RprY–LacZ fusion protein was induced specifically by Na^+^ depletion; however, *P. gingivalis rprY* and *oxyR* mutants were unable to grow in a similar medium. Microarray-based comparative transcription profiling of *P. gingivalis* parent and *rprY* mutant strains grown under Na**^+^** replete and depleted conditions confirmed the involvement of RprY in the oxidative stress response. These results were supported by EMSA assays that showed RprY binding not only to the promoter of alkyl hydroperoxidase, which detoxifies peroxide, but also to the promoters of several protein chaperones that protect against oxidative stress, i.e. *groES, clpB*, and *dnaK*. In addition, these experiments showed RprY bound to its own promoter, and so it was autoregulated.

As noted above, in *P. gingivalis* regulator RprY is an orphan. Indeed, the cognate HK, RprX, is present in all other oral and enteric Bacteroidetes except *P. gingivalis*, which does not contain proteins with any similarity to RprX. However, the gene adjacent to *rprY* encodes a protein acetyltransferase (*pat*: PGN_1185 in ATCC 33277); the *pat* and *rprY* genes have the same transcription start site and are co-transcribed [47]. Based on protein homology, Pat is a GCN5-related acetyltransferase (GNAT), proteins that were originally identified for their role in modification of eukaryotic histone proteins; however, recently, similarly acetylated proteins were identified in prokaryotes [48,49]. *N* ^ε^-Lysine acetylation of proteins is effected by acetyl CoA synthase and a GNAT that uses acetyl CoA as the acetyl donor with the concomitant release of CoA [50]. Acetyl CoA can be derived from pyruvate or from acetate via phosphorylation by acetate kinase (AckA); acetate phosphate is then converted to acetyl CoA by phosphotransacetylase (Pta). An interesting and potentially evolutionary adaptation is that in *P. gingivalis*, the genes for AckA and Pta are just upstream from those for Pat and RprY. This is not the case in oral Bacteroidetes that contain the *rprX* gene.

The acetylation modification is reversed by a deacetylase, and the bacterial CobB sirtuin was identified as responsible for this function [51]. Based on protein homology, we identified CobB (PGN_004) with 58% exact and 73% positive identity to the CobB protein from *Bacteroides thetaiotaomicron* (Accession number NP_811887). The findings that *pat* and *rprY* had the same transcriptional start prompted a study to determine whether acetylation, a post-translational modification, altered the regulatory functions of RprY in strain ATCC 33277 [47]. Western blots of cell extracts probed with anti-acetyl-lysine or anti-RprY antibodies showed that the parent strain contained acetylated RprY, which was absent in *rprY* mutant extracts. In addition, RprY could be chemically acetylated *in vitro* in a reaction that was dependent on acetyl CoA as the acetyl donor and recombinant Pat; the latter was not self-acetylated. Based on these observations, it was hypothesized that Pat functioned as the modifier of RprY protein *in vitro*. The results also suggested that acetylated RprY does not transfer acetyl groups to other proteins, indicating that it does not possess acetyltranferase activity. CobB, PGN*_*0004 in ATCC 33277, is a Sir2 family nicotinamide adenine dinucleotide (NAD^+^)-dependent deacetylase, and the acetylation level of RprY was significantly reduced in the presence of CobB and NAD^+^, while nicotinamide, a CobB inhibitor, reduced CobB-dependent deacetylation of RprY. Not surprisingly, the data obtained from electromobility shift assays (EMSA) indicated that acetylated RprY showed reduced binding to the promoter of *nqrA*, and addition of the CobB deacetylase restored binding. On the other hand, the presence of increasing concentrations of AcP (phosphate donor) resulted in increased incorporation of the phosphorylated protein into RprY–DNA complexes. Consistent with these data, *in vitro* chromatin immunoprecipitation (ChIP) assays to quantify binding of non-acetylated and acetylated RprY to sonicated genomic DNA fragments of *P. gingivalis* showed that binding of DNA to acetylated RprY was reduced approximately twofold compared with the unacetylated RprY control, while phosphorylation of RprY increased DNA binding at least threefold compared with the control. Thus, the DNA-binding ability of RprY was regulated by both acetylation and phosphorylation with the potential to alter the expression of virulence genes.

The relationship between acetylation of RprY and gene expression under Na**^+^**-depleted conditions was investigated [47]. Compared with extracts from cells from normal medium, those from Na**^+^**-depleted medium contained 60% less RprY, which was highly acetylated. To determine whether the repressor function of RprY was impaired in ATCC 33277 parent cells harvested from Na**^+^**-depleted medium, the relative expression of two promoter targets of RprY, i.e. *nqrA* and *rprY* itself, was measured. By QRT-PCR, expression of the *rprY* transcript was reduced in cells from Na**^+^**-depleted medium, consistent with the Western data, while expression of *nqrA* increased almost sevenfold, consistent with expression in the *rprY* mutant. Taken together, these results suggest that RprY may be a constitutively expressed repressor and that, in the absence of a cognate HK, its function is modulated by the acetylation activity of Pat. The exact role of Na^+^ depletion on expression of RprY is still unknown, although the *rprY* mutant has the same growth phenotype as an *oxyR* mutant during depletion, suggesting a role in the oxidative stress response [46]. Interestingly, it was recently demonstrated that the NQR pump of *V. cholerae* is a generator of ROS, specifically associated with reduced a flavin adenine dinucleotide cofactor of subunit F (*nqrF*) of the complex [45].

## haeSR system

Iron is an essential nutrient for survival of *P. gingivalis*, and the majority of iron in the human body is stored in haemoglobin as haem (iron complexed with protoporphyrin IX). *P. gingivalis* has a number of mechanisms for acquiring haem from haemoglobin and other host proteins such as degradation and haem binding by gingipains, followed by transport into cells by outer membrane receptors with a high affinity for haem. Preliminary *in vivo* ChIP-on-chip experiments established that the TCS encoded by PGN_0752-0753 (strain ATCC 33277) and PG0719-0720 (strain W83) was involved in haemin/iron acquisition because immunoprecipitates with anti-regulator (PG0720) antibody enriched for promoters associated with haemin transport. Therefore, the TCS was named HaeRS (for haemin). A mutant in the PGN_0752 HK (*haeS*) from ATCC 33277 could not be generated using DNA primers designed from the W83 genome sequence, and in a previous study it was noted that the genomic region surrounding this TCS was highly divergent between the strains [52]. Following publication of the genome sequence of ATCC 33277 [53], a comparison of these regions revealed a 2.529 kbp deletion in ATCC 33277 at the locus homologous to *haeSR* (PG0719-720) in W83. Thus, ATCC 33277 is a naturally occurring mutant in the TCS. Correspondingly, expression of the HKs and RRs was dramatically reduced, resulting in slower growth of the strain, but transfer of the functional *haeS* (PG0720) from strain W83 restored expression of the TCS and growth in the ATCC 33277 transconjugant [54].

Based on ChIP-seq data, HaeR binds at least two classes of promoters, depending on the specific concentrations of haemin used in growth media. In the first class, the number of bound HaeR sequences increased with the haemin concentration, implying that expression of these genes was induced by haemin. The gene cluster *hmuYRSTUV* is the best-characterized haemin transport system in *P. gingivalis*. Among the targets of HaeR identified by ChIP-seq were 5ʹ untranslated sequences upstream of PGN_0704 (*ihtA*), PGN_0687 (*htrA*) and PGN_0556 (*hmuS*). RR binding to these sequences was confirmed by EMSA, so we conclude that the three loci, consistent with their previously established roles in haemin transport, are directly regulated by PGN_0753 [55–57].

In the second class of HaeR promoter targets, the number of bound promoter sequences decreased in cells grown with increased haemin concentration, suggesting haemin repressed expression of the corresponding genes. Included in this class are several TonB-dependent receptors that transport haem across the outer membrane, and ABC transporters carry haem across the periplasm and inner membrane [58]. Gingipain cysteine proteinases comprise an important class of proteins that play a role in haem acquisition by releasing and binding haem from haemoglobin and other host proteins. The ChIP-seq data show that the promoter regions of RgpA and Kgp were enriched under haem-deficient and -limiting conditions, and HaeR bound to the promoter regions of both genes, indicating direct regulation. The first indication of Kgp involvement in haem accumulation came from a key genetic study showing that *kgp* mutant colonies did not present the normal black-pigmentation phenotype owing to haem adsorption at the cell surface [59]. Subsequent work showed that both proteinases were responsible for the capture of haemoglobin, its degradation and release, and conversion of haem to m-oxo bishaem aggregates [60,61]. Most recently, gingipain and HmuY activities have been linked together in the release of haem from proteins degraded by gingipains and its capture by HmuY [62].

In summary, the HaeR regulon includes a number of iron uptake/acquisition genes that encode transporters and metabolic functions, and HaeR acts as an activator or repressor depending on the target gene and the haemin concentration in growth media. Collectively, the ChIP-seq data suggest that the TCS is induced by low concentrations of haemin as indicated by increased expression and binding of HaeR to promoters in haemin-depleted or -limited conditions. Finally, it was established that the HaeSR regulon includes, and HaeR directly regulates expression of Kgp and RgpA, multifunctional virulence factors of *P. gingivalis.*

Genes associated with haemin acquisition predominated in the HaeR regulon, raising the question of whether haemin was the environmental signal that HaeS senses in order to activate the system. Because *P. gingivalis* ATCC 33277 is a naturally occurring *haeS* deletion strain, the environmental signal for HaeS was defined in an ATCC 33277 chimeric strain, TR719, which expresses the complete *haeS* gene (PG0719) from W83 and restores function to the HaeSR system of ATCC 33277 [54]. HaeS is a 427-amino-acid sensor kinase protein containing a conserved DHp (dimerization and histidine phosphotransfer) domain with H226 as the predicted phosphorylation site, an HATPase-c domain, and the periplasmic sensor region is 104 amino acids in length. Two *in vitro* approaches established that haemin bound to both the periplasmic and cytoplasmic domains of HaeS, but not to HaeR, the negative control [63]. Haemin bound to the HaeS dimer (101 kDa) *in vivo* after purification with haemin-conjugated agarose. The periplasmic domain of HaeS contains seven tyrosine residues, five of which are conserved in HKs of species most closely related to *P. gingivalis*, and two non-conserved histidine residues. Histidine and tyrosine residues bind iron and haemin with high affinity, and a point mutation Y88A showed a significant reduction in haemin binding.

HaeS is a bifunctional HK predicted to phosphorylate and dephosphorylate its cognate RR [12] and belongs to a subfamily that contains a specific and highly conserved amino acid motif, E/DxxN/T, adjacent to the phospho-accepting histidine H226. The E/D residue is required for kinase and N/T for phosphatase activities, respectively [64,65]. Experimentally, it was demonstrated that the recombinant cytoplasmic fragment of HaeS uses ATP as the phosphodonor to autophosphorylate the conserved histidine 226 residue, while a recombinant cytoplasmic fragment of HaeS carrying the H226A point mutation was not phosphorylated. The cytoplasmic domain of HaeS also contains the conserved ExxT motif associated with phosphatase activity as demonstrated *in vitro* by the ability of this domain to reduce phosphorylation of HaeR-P [63]. Thus, the cytoplasmic fragment of HaeS behaves as a phosphatase and dephosphorylates HaeR, and thus has the potential to modulate the function of the regulator. It is proposed that extracellular haemin binds with high affinity to the periplasmic domain of HaeS under haemin-limited conditions activating production of enzymes and transporters to acquire more haemin from host proteins. Haemin binds with low affinity to the cytoplasmic domain of HaeS under haemin-replete conditions allowing tighter regulatory control over the phosphorylation site and therefore activity of HaeR.

## PorXY

*P. gingivalis* is the most-studied anaerobe in the oral cavity, in part because they produce gingipains, a class of cysteine proteinases that play important roles in the nutrition, physiology, and virulence of the organism (reviewed in [66]). The study of these enzymes led to advances in the development of growth conditions, genetic systems, and tissue and animal models of infection for the bacterium. Arg-gingipains cleave proteins after arginine residues (RgpA and RgpB), and lys-gingipain (Kgp) cleaves after lysine residues. While RgpB (81.2 kDa) contains only catalytic activity, RgpA and Kgp are larger proteins (108.8 and 186.8 kDa, respectively) that possess N-terminal catalytic activities as well as extensive C-terminal adhesin domains that contain significant regions of sequence homology within individual peptides of the RgpA and Kgp adhesin domains. Gingipains are also associated with the black colony phenotype of *P. gingivalis* grown on blood plates, and after screening a *P. gingivalis* transposon library, a non-pigmented mutant was obtained in the *porT* gene that produced but was unable to secrete gingipains [67,68,69]. In addition to understanding the biochemistry and numerous functions associated with gingipain components, a key question has been: how are these large proteins secreted? Genomic analyses revealed that proteins related to PorT were associated with gliding motility in other species of Bacteroidetes, and mutant studies identified up to 10 orthologous and unique *P. gingivalis* genes associated with gingipain secretion by the Por Secretion System [70], later renamed the type nine (IX) secretion system because of conserved amino acid sequences characteristic of those secreted by the type IX system, present in gingipains and other *P. gingivalis* proteins [69]. The development of this research is comprehensively described in a recent review [66].

The PorXY genes have been identified as the TCS that regulates the type IX secretion system in *P. gingivalis*, whereby PorX (PGN_1019 in ATCC 33277 and PG0928 in W83) is the RR, and PorY (PGN_2001 in ATCC 33277 and PG0052 in W83) is the HK [69]. These authors reported that ECF SigP (PGN_0274 or PG0162), previously identified as associated with the production of gingipains [71,72], was also part of the PorXY TCS and directly interacted with the PorX RR. In another study, electromobility shift and DNA-protein co-purification assays showed that PorX does not bind directly to promoters of gene products that use the type XI secretion pathway, but rather binds to the cytoplasmic domain of PorL, part of the transmembrane complex. The authors suggest that PorX plays a role in the molecular machinery of secretion rather than direct regulation [73].

## GppX

*P. gingivalis* contains a naturally occurring HK–RR hybrid protein, called GppX, PGN_1768 or PG1797, because it was associated with gingipain production and localization, and consequently pigment production [74]. A *gppX* mutant appeared to produce less arg- and lys-gingipain than parent cells, and most of that was released into the culture medium rather than retained inside or at the cell surface. Another study suggested that GppX acted as a repressor, since production of LuxS was upregulated in a *gppX* mutant [75]. A more comprehensive study of the GppX regulon comparing the tanscriptomes of *gppX* parent and mutant strains was carried out using RNA-seq [76]. An interesting finding was that the *gppX* mutation impacted expression of nearly half the genes that encoded hypothetical proteins. For example, transcription of hypothetical PGN_ 0151 was downregulated in the *gppX* mutant, and the regulator bound directly to the promoter of upstream gene PGN_0152, which is co-transcribed with PGN_0151 and activated transcription. Clearly, studies with GppX and *P. gingivalis* are at an early stage, but recent reports defined candidate functions and a gene target for the orthologue of GppX (TF0022) in the oral anaerobe *T. forsythia* [77]. Disruption of the gene led to bacterial aggregation in broth culture, and changes in the 2-D proteome profile of the mutant compared with the parent strain were observed; specfically, a glycosyltransferase (TF1061) was affected. This gene is part of a cluster that also contains an AmpG permease involved in transport of degraded murein peptides into cells. Well known for its fastidious growth requirement for *N*-acetylmuramic acid, *T. forsythia* scavenges this muropeptide precursor produced by other bacteria during cell-wall recycling. A recent report demonstrated that expression of the operon containing the *ampG* gene was downregulated in the *gppX* mutant, confirming a nutritional function for the regulator in *T. forsythia* physiology, and perhaps a larger role in cell-wall biogenesis [78].

## Degeneracy of TCS in *P. gingivalis*

As suggested earlier, it is claimed that bacteria residing in secluded niches have fewer TCSs than those that grow in more diverse environments or have a complicated developmental cycle. This is an interesting issue for debate, and the obvious argument is that bacteria in every environment have evolved to optimize their fitness for survival. In *P. gingivalis*, each TCS studied in depth is somewhat atypical. As reported above, strain-specific mutations occurring in both FimRS and HaeSR render them non-functional or impaired. Therefore, studies comparing multiple strains such as that recently reported by [79] provide a valuable resource to discover more strain-specific differences that could impact infection.

## TCSs of oral streptococci

The complex and heterogeneous group of oral streptococci is currently classified into five groups: (1) Mutans, (2) Salivarius, (3) Anginosus, (4) Sanguinus, and (5) Mitis [80]. These groups diverge in abundance, preferred sites of colonization, and expression of functions associated with oral and systemic infections, including dental caries, bacteraemia, and infectious endocarditis. Species that predominantly initiate colonization of the supra-gingival dental surfaces under healthy-associated conditions include the Mitis (*Streptococcus mitis* and *Streptococcus oralis*) and the Sanguinus groups (*Streptococcus sanguinis* and *Streptococcus gordonii*) [81,82]. Although *S. mitis* and *S. sanguinis* are pioneer colonizers of teeth, *S. sanguinis* is mostly restricted to dental surfaces, while *S. mitis* is abundant in a large range of mucosal and tooth sites, and may persist in significant numbers in biofilms associated with caries [83,84], a consequence of the high level of genetic heterogeneity within the species [85,86]. On the other hand, Mutans species *S. mutans* and *S. sobrinus* show a low abundance in health-associated biofilms and are poor initiators of tooth colonization [82], but predominate in biofilm communities in the presence of sucrose and acidic environments promoting the growth of the acidogenic and aciduric microbiota associated with dental caries [14,87,88]. Sequence homology analyses reveal that different streptococcal species including *S. gordonii*, *S. sanguinis*, and *S. mutans* share several TCSs controlling functions for bacterial persistence in the oral cavity [87,89]. However, molecular studies indicate that even highly conserved TCS, e.g. TCS VicRK, have species-specific functions in bacterial colonization and virulence as addressed below [90,91].

Clinical, animal, biochemical, genetic, and molecular studies on *S. mutans*, the major species affecting host-biofilm homeostasis during caries development, revealed a panel of virulence genes for the synthesis of highly stable water-insoluble glucan from sucrose (*gtfB*, *gtfC*) and other exopolysaccharides (*ftf*, *gtfD*, *epsC*), for bacterial binding to these polymers (*gbpA*, *gbpB, gbpC*), and for production and tolerance to acids from fermentable sugars and other stresses (including multiple metabolic and stress response genes), as addressed in several reviews [8,9,92–94]. Some of these gene functions are also associated with the ability of *S. mutans* to avoid host immune functions in systemic infections [95]. However, expression of these genes is not constitutive, and expression under different conditions varies between strains [95–97]. Increased expression of virulence functions was observed in strains associated with cariogenicity and systemic diseases (e.g. bacteraemia and infectious endocarditis) compared with strains isolated under healthy conditions [95,97]. Therefore, it is necessary to define the TCS regulatory circuits in *S. mutans*, as well as in other streptococcal species in oral biofilms in order to understand the environmental signals that trigger expression of virulence phenotypes. The large number of TCSs expressed by most oral streptococci (Table 1) poses a challenge to understand these complex regulatory functions, in part because multiple TCSs are coordinately activated in response to stimuli of specific growth conditions. However, interesting findings have been obtained by analysing TCSs that are highly conserved across streptococcal species, including the VicRK and CovRS TCS, which play important functions for coordinating bacterial growth with virulence functions for persistence in host sites.

## TCS VicRK of streptococci

**Figure 2.**
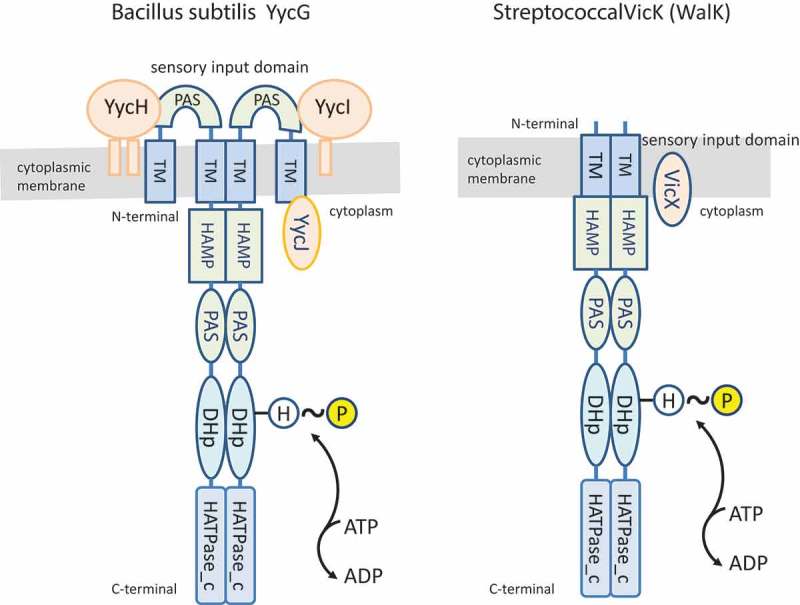
Comparison of the domain architecture of histidine kinases of the VicRK TCS. The *B. subtilis* VicK orthologue (known as YycG) has an extracellular loop in the sensory input domain that includes an accessory PAS domain. YycG is also associated with ancillary proteins YycH, YycI, and YYcJ, encoded by the five-cistron operon *yycFGHIJ*. A similar operon structure is found in most genera of *Firmicutes*, including *Staphylococcus* spp., *Listeria* spp., *Enterococcus* spp., and *Lactobacillus* spp. The streptococcal VicK is expressed from a three-cistronic *vicRKX* operon, which includes a single accessory cistron encoding VicX (an orthologue of YycJ), the VicR RR (*vicR*), and the histidine kinase VicK (*vicK*), also known as WalK. The streptococcal VicK lacks the extracellular loop in the sensory input domain. The cytoplasmic transmitter domains of the VicK and YycG histidine kinases share a similar domain architecture, which includes the accessory HAMP, PAS, DHp, and HATPase_c domains. The cognate RRs YycF of *B. subtilis* and *VicR* of streptococci are not shown in these schaems.

The TCS VicRK (also known as YycFG, and WalRK) is ubiquitous in the Firmicutes phylum, and one of the few TCSs essential for viability in several low GC Gram-positive bacteria. The system was first discovered in *Bacillus subtilis* as essential for cell division and growth because it directly regulates the *ftsAZ* operon [98,99]. Orthologues of *vicR* and *vicK* form operons with one to four accessory cistrons [100]. In *B. subtilis*, two of these cistrons (*yycH* and *yycI*) encode transmembrane proteins that negatively regulate YycFG (VicK) kinase activity, but the function of a third gene (*yycJ*) is still unclear [101]. A similar operon structure is found in most other Gram-positive genera, e.g. *Staphylococcus, Enterococcus*, *Listeria*, and *Lactobacillus*, except for *Streptococcus* spp. and *Lactococcus lactis*, in which the operons include only the *yycJ* orthologue, also known as *vicX* or *walJ* [100,102]. Proteins encoded by *vicX* are metal-dependent beta-lactamases of as-yet unknown function, although there is evidence suggesting that VicX modulates VicK activity in *S. mutans*, because *vicX*-defective strains showed altered phenotypes in VicRK-regulated functions [103]. VicX of *S. pneumoniae* is localized in the membrane, implying an interaction with the VicK sensor protein [102]. Figure 2 illustrates differences between *B. subtilis* and streptococcal species in the domain architecture of YycG (VicK) and ancillary proteins of the VicRK TCS (YycFG). In *B. subtilis* and other non-streptococcal species, the N-terminus of the HK includes two TM domains flanking an extracellular sensor loop, while the streptococcal VicK proteins have a single TM linked to a short extracellular peptide [102]. In a model for function of YycFG and associated ancillary proteins, an extracellular signal activates YycG by affecting inhibitory interactions of YycI and YycH. The lack of the extracellular loop of streptococcal VicK plus the absence of YycI and YycH orthologues suggests that VicK is activated by stimuli at the cytoplasmic membrane in a process involving VicX. As with YycG, VicK has two intracellular accessory domains, HAMP and PAS, next to the TM region. HAMP and PAS domain functions are not completely understood [101,104], although PAS domains respond to input signals including redox potential, oxygen, and small ligands, which could cross the cell membrane or affect cell metabolism [101,105].

**Figure 3.**
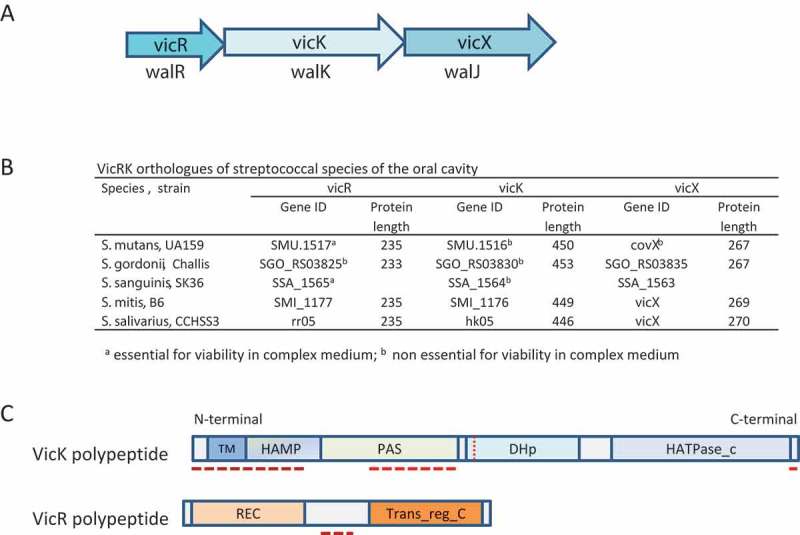
VicRK orthologues of oral streptococcal species. (a) Coding regions of the genes of the *vicRKX* operon represented by arrrows indicating the direction of transcription; orthologous genes found in streptocccal species are also called *walRKJ*. (b) Genes and protein length of VicRKX orthologues found in different species of oral streptococci. Gene ID numbers were obtained from GenBank (https://www.ncbi.nlm.nih.gov/gene). (c) Schematic representation of domain architectures of VicK and VicR polypeptides (coloured bars), which were determined using the SMART database (http://smart.embl-heidelberg.de/). Transmembrane (TM), HAMP, PAS, DHp, and catalitic HATPase_c domains are indicated within each coloured module. ClustalW multiple sequence alignment (http://www.genome.jp/tools-bin/clustalw) of amino acid sequences of streptococcal VicK and VicR proteins listed in Table B revealed regions with sequence diversity between species, as indicated by dashed red lines.

Figure 3 shows the structure of *vicRKX* operons and protein similarities in pathogenic and commensal species of oral streptococci. Analyses of VicK sequences using the SMART program detected a HAMP domain in *S. gordonii* and *S. salivarius*, but not in *S. mutans*, *S. sanguinis*, or *S. mitis*, reflecting low conservation of this domain. In addition, the N-terminus of VicK and part of the PAS domain are less conserved among species (Figure 3(c)). Whether diversity in these variable regions accounts for functional differences between streptococcal species remains to be investigated. VicR is highly conserved among streptococcal species with polymorphic sequences restricted to linker regions between receiver and output domains. VicR is in the OmpR/PhoB family of regulators in which the output domain forms a DNA-binding winged-helix domain. In *B. subtilis* and *Staphylococcus aureus*, both VicR and VicK are essential for bacterial viability [100]; on the other hand, only *vicR* is essential in *S. pneumoniae* [105], *S. mutans* [106,107], and *S. sanguinis* [90], and both *vicR* and *vicK* can be deleted in *S. gordonii* [91]. In *S. pyogenes*, insertional inactivation of *vicR* was possible, but the mutant was not viable in a mice model of infection [108]. The exact reasons for VicR and/or VicK essentially in different species are unknown, but a possibility is their role in the regulation of peptidoglycan (murein) biosynthesis, cell division and cell wall homeostasis [90,98,103,107,109–111]. Because *vicK* can be deleted in *S. pneumoniae, S. mutans*, and *S. sanguinis*, one possibility is that the VicR of these species could be phosphorylated by alternative pathways.

## VicRK regulons of TCSs are diverse and species-specific

VicRK plays an important role in modulating cell division, cell-wall biosynthesis and homeostasis, and membrane integrity, as evidenced by the presence of several genes encoding autolysins and other murein hydrolases in the VicR regulons of *B. subtilis* [112], *S. aureus* [109], *S. pneumoniae* [105], *S. mutans* [107,113], and *S. sanguinis* [90]. Consistently, atypical long-chain phenotypes are found in v*icK* isogenic mutants obtained in *S. pnemoniae*, *S. mutans*, and *S. sanguinis* [90,105,106,111]. In VicR regulons, the most conserved target gene encodes PcsB (protein required for cell separation of group B *Streptococcus*) that was first identified in group B streptococci [114] and orthologue GbpB (glucan-binding protein B in *S. mutans*) [115,116]. Table 2 shows comparisons of VicR gene targets among streptococcal species of the oral cavity and oropharynx. PcsB/GbpB are cell-surface murein hydrolases with CHAP amidase domains and are part of the divisome, at least in *S. pneumoniae* [119–122]. PcsB/GbpB was implicated in the essentiality of VicRK in *S. pneumoniae*, where *vicR* deletion is possible only when *pcsB* is induced [119]. In *S. mutans*, GbpB/PcsB is required for surface binding to glucan exopolysaccharides synthesized from sucrose during biofilm formation [111].

**Table 2.** Direct VicR gene targets that are positively (+) or negatively (–) regulated in different species of streptococci of the oropharynx.

		Streptococcal species				
Gene name	Encoded protein or function	*S. mutans*	*S. sanguinis*	*S. gordonii*	*S. pneumoniae*	References
Cell-wall biogenesis, division, homeostasis						
*gbpB/pcsB*	Glucan-binding protein B (GbpB)/protein for cell separation of group B streptococcus (PcsB)	(+)	(+)	ND	(+)	[90,105,111,117]
*smu.2147c*	LysM; murein hydrolase	(+)	WO	WO	WO	[107]
*smu.2146c*	Putative murein hydrolase	(+)	WO	WO	WO	[107]
*smaA*	Murein hydrolase	(–)	WO	WO	WO	[107]
*smu.367*	Putative murein hydrolase	(+)	WO	WO	WO	[107]
*cwdP*	Putative murein hydrolase	WO	(+)	ND	WO	[90]
*SSA_0094*	Putative murein hydrolase	WO	(+)	WO	WO	[90]
*Smu_689 (atlA)*	Autolysin	(+)	WO	WO	WO	[117]
Synthesis of exopolysaccharides from sucrose						
*gtfB*	Glucosyltransferase B	(+)	WO	WO	WO	[106]
*gtfC*	Glucosyltransferase C	(+)	WO	WO	WO	[106]
*ftf*	Fructosyltransferase	(+)	WO	WO	WO	[106]
Competence						
*comCDE*		(–)	(–)^b^	ND	(–)^c^	[90,110,113]
Copper homeostasis						
*copY*	Copper-responsive repressor	(–)	ND	ND	ND	[117]
Production of bacteriocin						
*nlmC*	Mutacin V	(+)	WO	WO	WO	[113]
Transcriptional regulation						
*covR* (*gcrC*)	Response regulator CovR	DB^a^	ND	ND	ND	[117]
Production of H_2_O_2_						
*spxB*	Pyruvate oxidase	WO	(+)	ND	ND	[90]
Evasion to complement immunity						
*pspA*	Pneumococal surface protein A	WO	WO	WO	(+)	[105]
*pepO*	Endopeptidase O	(–)	ND	ND	ND	[118]
*smu.399*	Putative C3-degrading proteinase	(+)	WO	WO	WO	[118]

Orthologous proteins were considered as those showing ≥50% identity in the amino acid sequences over at least 80% of the length of the shortest protein.

WO: without orthologous gene/protein. ND: not determined. DB: evidence of VicR binding to the promoter region, without transcriptional analysis.

^a^ No direct binding to *covR* promoter region observed in EMSA assays [107].

^b^ Significant transcriptional changes of *comE* at late(–)log phase of growth; no evidence of direct VicR binding [90].

^c^ Significant transcriptional changes in *comE* promoted by *vicRKX* downregulation in *S. pneumoniae*; no evidence of direct VicR binding [110].

In addition to GbpB/PcsB, several other non-essential murein hydrolases are directly regulated by VicR in *S. mutans*, including SMU_2146c, SMU.2157c (LysM), SmaA, SMU.367 [107], and the autolysin A (AtlA; smu_689) [117,123]. However, these proteins are species-specific. Similarly in *S. sanguinis*, the VicRK*_Ss_* regulon includes genes for murein hydrolases SSA_0094 and SSA_0304 that are not expressed by *S. mutans* [90]. Most genes directly regulated by VicR in *S. mutans* are not present in the genomes of other streptococci and vice versa (Table 2). The *S. mutans* VicRK also induces expression of GtfB and GtfC, which are required for the synthesis of extracellular insoluble glucan [106,107] the major extracellular matrix component of *S. mutans* biofilms [124,125]. GtfB and GtfC are not expressed by commensal *S. sanguinis* or *S. gordonii*, although these species express Gtfs that synthesize different types of glucan [125]. The expression of glucan-binding proteins allows *S. mutans* to bind to glucan in biofilms, a mode of growth that affords evasion of complement [95], an important host-defence system present in saliva, GCF, and blood [126,127]. In *S. mutans*, VicR*_Sm_* negatively regulates *pepO* and *SMU*_*399*, which encode proteases implicated  in evasion of complement system [118]. In addition, VicRK*_Spn_* regulates *pspA* [105], which encodes a species-specific surface protein (PspA) involved in complement evasion [128]. In *S. mutans*, VicR*_Sm_* also directly represses genes involved in competence and competence-induced bacteriocin (*comCDE* and *nlmC*) [113], and *copY* [117], part of the *copYAZ* operon required for copper homeostasis and regulation of membrane potential, competence, and biofilm formation [129]. In *S. sanguinis*, VicRK*_Ss_* does not regulate *gtfP*, a unique gene encoding a glucosyltransferase for the synthesis of soluble glucan [90] but does regulate *spxB* encoding pyruvate oxidase, required for conversion of pyruvate, inorganic phosphate, and O_2_ to H_2_O_2_, CO_2_, and AcP [130]. Production of H_2_O_2_ by *S. sanguinis* inhibits the growth of the competitor species *S. mutans* [131]. By mechanisms not completely understood, H_2_O_2_ is also required for release of DNA (eDNA) to the extracellular milieu [132], where it is a major component of the extracellular matrix of *S. sanguinis* biofilms [90]. Thus, the VicRK regulons include species-specific genes involved in bacterial persistence in host niches, including immune evasion, inter-species competition, and biofilm formation.

## What are the signals sensed by VicRK TCS?

The general role of VicRK in controlling the biosynthesis of peptidoglycan and cell division might imply that this TCS senses signals associated with peptidoglycan biosynthesis [100]. In *B. subtilis*, YycG (VicK) co-localized with FtsZ of the divisome, and it was proposed that VicK*_Bs_* senses cell division directly [133]. *S. pneumoniae*, VicK*_Spn_* does not co-localize with the FtsZ ring at division septa, but is peripherally distributed during exponential growth [102]. However, VicRK*_Spn_* target protein, PcsB, localizes at the cell-division septa by interacting with FtsX, a component of the divisome [102,121]. It is possible that signals sensed by human streptococci are different from those that activate YycG of *B. subtilis*, present in soil and water habitats, microbial communities of plants, and gastrointestinal tracts of animals [134]. The domain architecture of streptococcal VicRK associated with molecular studies is compatible with the hypothesis that VicRK could be activated by stresses to the cell envelope that would promote changes in redox potentials sensed by the cytosolic PAS domain. In *S. mutans*, transcripts of *vicRKX* are found during the mid- and late-exponential phases of growth, but reduced during early-exponential growth [96,135]. Furthermore, *vicR_Sm_* transcription is induced by vancomycin and other antibiotics that target peptidoglycan biosynthesis (ampicillin, penicillin G) or the cell membrane (polymyxin B) [135]. Increased transcript levels of *vicR_Sm_* are observed during initial phases of biofilm growth in the presence of sucrose compared with planktonic cells (at mid-exponential growth) or to cells of biofilms formed in the absence of sucrose [107]. These findings suggest that VicRK*_Sm_* is activated not only by intrinsic peptidoglycan growth and cell division, but also by processes associated with sucrose-dependent biofilm initiation. Consistent with these data, VicR*_Sm_* directly induces expression of enzymes required for the synthesis of different sucrose-derived exopolysaccharides (GtfB, GtfC, Ftf) and glucan-binding (GbpB) [106,111,117].

To explore signals activating VicK in *S. pneumoniae*, strains expressing truncated versions of *vicK* were constructed to identify domains involved in VicK kinase activities. These studies revealed that VicK is a bifunctional sensor protein that alternates kinase and phosphatase activities, and that the phosphatase activity is dependent on the PAS domain [102,136]. There is also evidence that both forms of VicR, phosphorylated (P-VicR) and non-phosphorylated (NP-VicR), are capable of directly binding to a different set of target genes. P-VicR induces expression of PcsB and other murein hydrolases, but NP-VicR directly represses *fabT*, encoding a repressor of genes for fatty acid biosynthesis and, in turn, upregulates membrane synthesis [137]. Phosphatase activity has also been shown in the *S. aureus* VicK, also known as WalK [138]. Therefore, VicRK TCS apparently switches functions affecting the homeostasis of the cell envelope both by its kinase and phosphatase activities, and, in turn, by balancing the levels of P-VicR and NP-VicR. These findings would imply that streptococcal VicRK is constantly active, and perhaps its kinase and phosphatase functions are modulated by stimuli affecting cell growth and cell-envelope homeostasis.

## Interactions of VicRK TCS with other regulatory systems

Bacteria inhabiting complex environments, such as the oral cavity, must integrate responses to a range of environmental stimuli [31]. Such integrated responses imply a certain degree of cross-talk. There is evidence that VicRK interacts with other regulatory circuits involved in cell-envelope homeostasis during challenges with a range of environmental stresses [107,138,139]. Some of the regulators found to interact or to cooperate with regulation of VicRK-gene targets include the orphan regulator CovR of *S. mutans* [107,117] and the TCS SaeSR of *S. aureus* [138]. In *S. mutans*, *vicRK* expression seems to be also indirectly induced by the TCS LiaSR [135], a TCS that regulates responses to cell-envelope stresses [140].

In *S. mutans*, there is a clear overlap between the regulons of VicRK*_Sm_* and of CovR*_Sm_*, the latter being an orphan RR in oral streptococci. The overlap includes genes for cell-wall division and biofilm formation (*gbpB*, *lysM, gtfB*, *gtfC*), with VicR positively regulating these genes and CovR acting as a repressor [107,141,142]. In EMSA assays, it was observed that VicR*_Sm_* VicR and CovR co-bind the promoter regions of *lysM*, *gbpB*, and *gtfC* [107]. CovR is also a regulator of functions required for evasion to host immunity [95], and VicR*_Sm_*/CovR*_Sm_* co-regulatd genes include those required for evasion of complement and opsonophagocytosis, i.e. *pepO* and *smu.399* [118,143]. These findings indicate that VicR/CovR cooperate to more precisely coordinate functions of cell division with biofilm formation and evasion of host immunity, two essential functions for *S. mutans* persistence in oral niches. In *S. aureus*, VicRK*_Sau_* interacts with the SaeRS TCS to upregulate expression genes with similar functions, and so it is possible that VicRK systems may cross-talk with other TCSs involved in bacterial evasion of host immunity [138]. Direct approaches to establish the molecular mechanisms underlying these potential cross-talks remain to be performed.

In addition to potential cross-talk between TCS, studies on streptococci and other Gram-positive bacteria indicate that VicKR can interact with a transmembrane eukaryotic-like serine/threonine kinase (STK) [144,145] that functions in concert with cognate cytoplasmic eukaryotic-like serine/threonine phosphatases (STP) present in Gram-positive bacteria as STK–STP operons [146,147]. STK are transmembrane enzymes with extracellular domains containing one to five PASTA (penicillin-binding protein and serine/threonine kinase associated domain) repeats and a cytoplasmic kinase domain [146,148]. PASTA domains sense unlinked muropeptides and localize the enzyme complexes for peptidoglycan biosynthesis [148]. In *S. pneumoniae*, STK*_Spn_* co-localizes with FtsZ at the division septum [149]. STK also binds elongation factor Tu (EF-Tu) [146], which could potentially explain the reported interaction of GbpB/PcsB with EF-Tu in *S. mutans* [122]. In *S. pyogenes*, STK*_Spy_* phosphorylates VicR*_Spy_* [145], and in *S. mutans*, deletion of the gene encoding STK (*pknB*) affected transcription of smu.2146c [144], a VicR target, which encodes a protein harbouring a lysozyme-like domain [107]. STK is conserved among Gram-positive bacteria, including oropharyngeal streptococci, e.g. *S. pneumoniae*, *S. gordonii*, *S. sanguinis*, and *S. mutans*, and analysis of these orthologues might help decipher the VicRK network [147]. Analysis of the STK-STP system in *S. mutans* was investigated using an isogenic mutant in *pknB*, the STK-encoding gene [144,150,151]. The mutant showed reduced cariogenicity in a rat model of dental caries, and defects in biofilm formation, competence, production of bacteriocins, and sensitivity to acid stress [144,150] and to H_2_O_2_ produced by *S. sanguinis* [151]. Although the molecular mechanisms underlying this phenotype remain to be investigated, it is compatible with the evidence that STK PknB activates VicR*_Sm_* in *S. mutans* [144]. The role of PknB in VicR phosphorylation might also explain the essentiality of *vicR*, but not of *vicK* in some oral streptococci, i.e. the sensor protein VicK might not be essential because VicR can be activated by PknB. Consistent with this hypothesis, it was not possible to obtain double mutants of *vicK* and *pknB* in *S. mutans* [144]. In addition to the roles of VicRK and STK-STP in bacterial responses to environmental stresses and host immune challenges, these systems also affect bacterial susceptibility to different classes of antibiotics. Accumulated mutations in *vicRK* and STK-encoding genes were detected among *Staphylococcus* strains resistant to vancomycin and daptomycin that target the cell wall and membrane, respectively [152–154]. Also, STK protein PknB (also called Stk1) appears to activate a transcriptional regulator of the antibiotic efflux pump NorA [146]. Further understanding of the molecular mechanisms involved in the interactions between these regulatory systems will increase interest in their potential as therapeutic targets to control infections.

## TCS CovRS, and CovR as an orphan regulator

The TCS CovRS was identified in beta-hemolytic *S. pyogenes* by screening transposon mutants for defects in genes in the *has* operon that are required for the synthesis of hyaluronic acid capsule. The TCS was originally designated CsrRS (Csr: capsule synthesis regulator) [155,156] and then renamed CovRS*_Spy_* (cov; control of virulence) based on the demonstration that the system repressed multiple virulence genes, including streptokinase (*ska*), streptolysin S (*sagA*), and mitogenic factor (*speMF*) [157]. Later studies showed that CovRS*_Spy_* directly or indirectly regulates approximately 10–15% of the *S. pyogenes* genome [158,159]. The TCS CovRS*_Spy_* is atypical because of its repressor functions, although it also positively regulates some genes in its regulon. Deletion of *covR_Spy_* increases transcription of a panel of CovR*_Spy_*-repressed genes and also survival of *S. pyogenes* to opsonophagocytic killing by human PMNs *in vitro* [155,158].

Only the orphan RR CovR is conserved in oral streptococci and in *S. pneumoniae* in which it is known as RitR (repressor of iron transport) [160]. In *S. mutans*, the system was originally designated GcrR (for glucan-binding protein C regulator) following identification in a screen for genes that regulate aggregation between GbpC and dextran [161]. Later studies revealed that CovR*_Sm_* negatively or positively regulates about 6.5% of the *S. mutans* genome [142]. More importantly, CovR*_Sm_* directly represses a panel of genes required for the synthesis of water-soluble and water-insoluble glucan (*gtfB*, *gtfC*) and other exopolysaccharides derived or not from sucrose (*epsC* and *ftf*), as well as surface proteins GbpC and GbpB that bind to glucan [107,141,142]. Deletion of *covR*_Sm_ reduced *S. mutans* cariogenicity in rat models by mechanisms not completely understood, but likely by affecting biofilm structure [162]. On the other hand, we recently showed that *covR*_Sm_ deletion increases *S. mutans*’s capacity to stably bind sucrose-derived exopolysaccharides mediated by GbpC and EpsC, and that this property provides a capsule-like protection against opsonophagocytosis and killing by human PMNs, as well as bacterial survival in blood [95]. *S. mutans* strains isolated from systemic infections show low levels of *covR*_Sm_ expression associated with derepressed transcription of CovR*_Sm_* target genes (*gbpB*, *gbpC* and *epsC*), and increased resistance to opsonophagocytic killing *in vitro*, supporting the important role of CovR*_Sm_* in the virulence of *S. mutans* [95]. CovR*_Sm_* was also shown to repress complement system proteases directly or indirectly, suggesting that, as for *S. pyogenes*, CovR*_Sm_* plays a role in host immune evasion and host invasiveness [143].

## Signals that activate CovRS or orphan regulator CovR

CovR*_Spy_* is an RR of the OmpR family, and CovS*_Spy_* is part of the EnvZ family of HKs. CovS*_Spy_* contains two N-terminal transmembrane domains flanking a predicted external input domain of 150 amino acids, as well as the intracellular HAMP, DHp and C-terminal HATPase_c domains. There is evidence that CovS*_Spy_* is a bifunctional sensor protein, switching kinase or phosphatase activities depending on the environmental stimuli. Under growth conditions, CovS*_Spy_* acts as a kinase that phosphorylates CovR at conserved apartate (D-53) of the receiver domain, thus increasing its affinity for target gene promoters leading to repression of transcription [163]. Under environmental stress conditions including high temperature (40°C), low pH (≈6.0), and high salt concentration, CovS*_Spy_* acts as a phosphatase that dephosphorylates CovR*_Spy_*, which derepresses transcription of target genes [164]. Interestingly, although dephosphorylation of P-CovR_Spy_ depends on CovS_Spy_, CovR*_Spy_* is still phosphorylated in *covS_Spy_*-defective strains, suggesting that CovR*_Spy_* is also phosphorylated by other TCS HK or additional kinases [164]. Because multiple stress conditions promote CovS*_Spy_* phosphatase activity, it was proposed that CovS_Spy_ is responsive to general stresses affecting membrane structure [164]. On the other hand, there is also evidence that CovS*_Spy_* activities are modulated by specific external ligands, including Mg^2+^, but not other divalent cations, and the human antimicrobial peptide LL-37 at subinhibitory concentrations, but not other antimicrobial peptides [165–168]. CovR*_Spy_* is also phosphorylated at the threonine residue T-65 by an STK [145,169], similar to CovR*_Sag_* of *S. agalactiae* [170]. Additional tyrosine/serine/threonine sites for CovR_Spy_ phosphorylation were also suggested [171] indicating complexity of mechanisms for activation of this RR.

**Figure 4.**
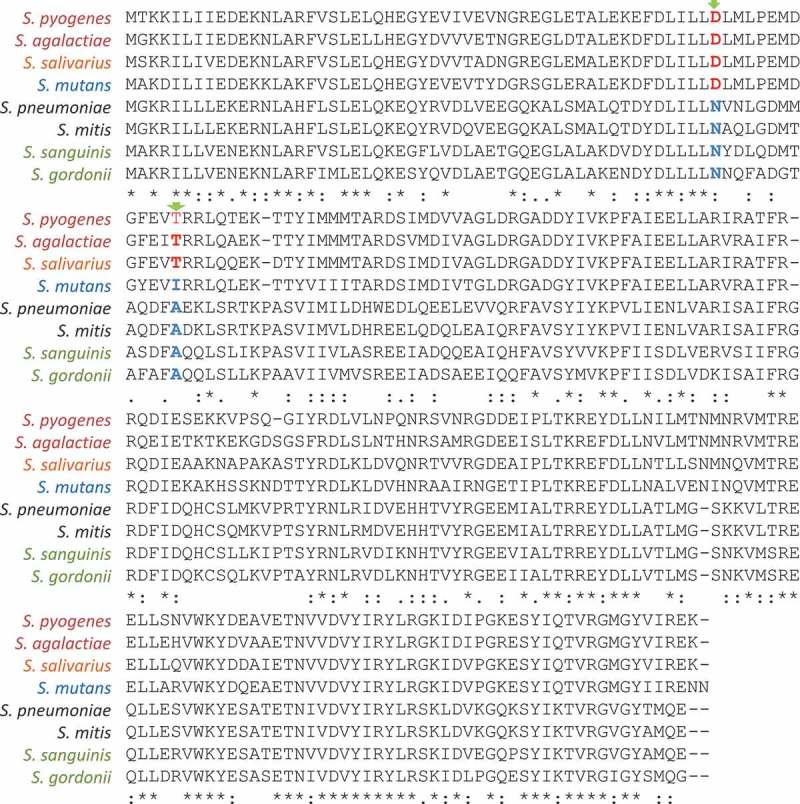
ClustalW multiple sequence alignment of the amino acid sequences of CovR orthologues of streptococcal species. Except for *Streptococcus salivarius*, CovR orthologues in oral streptococci and *S. pneumoniae* do not have the aspartate (D-35) and/or threonine (T-65) residues (marked in red) known to be phosphorylated by CovS histidine kinase and serine/threonine kinases, respectively, in the beta-hemolytic *S. pyogenes* and *S. agalactiae* species. The D-35 aspartate is conserved in *S. mutans* CovR. Sequence alignments were performed using the bioinformatics tool available at http://www.genome.jp/tools-bin/clustalw.

Studies on molecular mechanisms of CovR phosphorylation and activities in oral streptococci are few. Comparisons of the amino acid sequences of CovR orthologues between the beta-haemolytic species (*S. pyogenes* and *S. agalactiae*) with oral streptococci (Figure 4) revealed that *S. salivarius* CovR has both D-53 and T-65 phosphorylation sites. On the other hand, only the D-53 site is conserved in *S. mutans*, while neither the D-53 nor T-65 phosphorylation sites are present in CovR orthologues of streptococci of the Mitis and Sanguinus groups (Figure 4), implying different mechanisms of CovR activation between streptococcal species. In *S. mutans*, it was reported that *in vitro* phosphorylation of CovR*_Sm_* does not increase the binding affinity to target promoters [141]. It was proposed that CovR*_Sm_*-mediated regulation is dependent on the amounts of CovR*_Sm_* present in cells regardless of the phosphorylation state; i.e. CovR*_Sm_* would be always active and/or likely phosphorylated by small phosphodonors such as AcP [141,172]. The absence of CovS protein in *S. mutans* and amino acid differences between CovR*_Spy_* and CovR*_Smu_* [142] (Figure 4) account for this proposal. This hypothesis is compatible with our findings that *S. mutans* strains isolated from systemic infections that have increased resistance to opsonophagocytic killing by PMN express low levels of *covR_Sm_* and upregulated CovR*_Sm_* target genes compared with oral strains [95]. Mechanisms regulating *covR*_Sm_ expression and the CovR regulon remain to be investigated in *S. mutans* strains. We have preliminary data indicating that BHI supplementation with human serum (20%) significantly reduces *covR* transcription in *S. mutans* strain UA159 [Mattos-Graner et al., unpublished data] suggesting that host components could induce *covR_Sm_* downregulation and derepress transcription of genes for immune evasion such as *pepO* and *smu.399*, which are also regulated by the TCS VicRK [118]. Unpublished results reveal that *pepO* and *smu.399* are also upregulated in the *S. mutans covR*-defective strain, connecting the TCS VicRK and CovR in modulating *S. mutans* evasion to host components [143]. Further studies are required to investigate serum-mediated signalling pathways by which CovR and VicRK regulate *S. mutans* responses to host stimuli, and cause oral and systemic infections.

## Concluding remarks and future work

Evolutionary analyses of bacterial signal transduction systems suggest that the number of TCSs in each oral species correlates with genome size and content, and the complexity of the environmental sites to which they have become adapted.

Although oral Bacteroidetes species have far fewer TCSs than their enteric relatives, they play vital roles such as protection against environmental stress and acquisition of essential nutrients. Oral streptococci are equipped with a considerably larger panel of TCSs, of which many are highly conserved across species. However, TCS orthologues have species-specific functions, as revealed by significant differences in their regulons as well as polymorphisms of regulatory proteins. VicRK and CovR are prototypic TCSs involved in coordination of cell growth and cell-wall homeostasis with fitness and persistence in host environments during streptococcal infections. Thus, TCSs have a central role in oral ecology.

As in all scientific fields, more knowledge about TCS regulation leads to more questions. For example, what are the support mechanisms provided by additional regulatory proteins such as one-component systems and extracytoplasmic-function σ factors (ECFs), as well as regulatory RNA species? Why do some systems degenerate and compensatory functions evolve? On a more practical level, identifying the regulons of each TCS will identify functional specificities in different species that could lead to the development of therapies to control local or systemic infections. Studies are necessary to understand the role of streptococcal TCS in modulating commensal to pathogenic interactions with the host, and in inter-species interactions. Increasing evidence that streptococcal TCSs are activated in response to host immune factors emphasizes the need to investigate TCS functions under host-like conditions, for example in the presence of serum, saliva, blood, and other host components.
